# Health economic benefits through the use of diagnostic support systems and expert knowledge

**DOI:** 10.1186/s12913-021-06926-y

**Published:** 2021-09-09

**Authors:** Tina Willmen, Lukas Völkel, Simon Ronicke, Martin C. Hirsch, Jessica Kaufeld, Reinhard P. Rychlik, Annette D. Wagner

**Affiliations:** 1 Department of Nephrology, Hannover Medical School, Hanover, Germany; 2 Institute for Empirical Health Economics, Burscheid, Germany; 3 Medical Clinic for Nephrology and Internal Intensive Care Medicine, Charité Berlin, Berlin, Germany; 4 Institute for AI in Medicine, University Hospital of Giessen and Marburg, Marburg, Germany; 5 Ada Health GmbH, Berlin, Germany

**Keywords:** rare diseases, health economic costs, diagnosis support systems, artificial intelligence

## Abstract

**Background:**

Rare diseases are difficult to diagnose. Due to their rarity, heterogeneity, and variability, rare diseases often result not only in extensive diagnostic tests and imaging studies, but also in unnecessary repetitions of examinations, which places a greater overall burden on the healthcare system.

Diagnostic decision support systems (DDSS) optimized by rare disease experts and used early by primary care physicians and specialists are able to significantly shorten diagnostic processes. The objective of this study was to evaluate reductions in diagnostic costs incurred in rare disease cases brought about by rapid referral to an expert and diagnostic decision support systems.

**Methods:**

Retrospectively, diagnostic costs from disease onset to diagnosis were analyzed in 78 patient cases from the outpatient clinic for rare inflammatory systemic diseases at Hannover Medical School. From the onset of the first symptoms, all diagnostic measures related to the disease were taken from the patient files and documented for each day.

The basis for the health economic calculations was the Einheitlicher Bewertungsmaßstab (EBM) used in Germany for statutory health insurance, which assigns a fixed flat rate to the various medical services.

For 76 cases we also calculated the cost savings that would have been achieved by the diagnosis support system Ada DX applied by an expert.

**Results:**

The expert was able to achieve significant savings for patients with long courses of disease. On average, the expert needed only 27 % of the total costs incurred in the individual treatment odysseys to make the correct diagnosis. The expert also needed significantly less time and avoided unnecessary examination repetitions.

If a DDSS had been applied early in the 76 cases studied, only 51–68 % of the total costs would have incurred and the diagnosis would have been made earlier. Earlier diagnosis would have significantly reduced costs.

**Conclusion:**

The study showed that significant savings in the diagnostic process of rare diseases can be achieved through rapid referral to an expert and the use of DDSS. Faster diagnosis not only achieves savings, but also enables the right therapy and thus an increase in the quality of life for patients.

**Supplementary Information:**

The online version contains supplementary material available at 10.1186/s12913-021-06926-y.

## Introduction

In Europe diseases are considered rare when they affect fewer than 1 in 2.000 persons [1]. Internationally, however, different definitions are used in some cases. A disease is classified as rare in the USA if no more than 200.000 residents are affected across the USA [2]. It is estimated that 8.000 rare diseases are known worldwide today [3]. Because of their variability, complexity and the lacking knowledge about rare diseases it is very difficult for most physicians to quickly identify rare diseases [4]. 25 % of the patients with rare diseases wait between 5 and 30 years until they are diagnosed correctly [5].

Surveys conducted in the USA and Great Britain revealed that the average patient visits eight physicians, four outpatient departments and four specialists until the correct diagnosis is found while getting two to three false diagnoses on the way to it [6].

A further problem is that there is often no interdisciplinary exchange between the treating physicians who see the patient in the course of their diagnostic odyssey [7].

Extensive tests and imaging examinations are thus unnecessarily repeated resulting in a delay in treatment and as a consequence in higher health care costs [7]. Compared to frequent and rare diseases cost bearers in the USA report up to 100 % higher costs for rare diseases [6]. Seen in international terms, the long road to diagnosis does not only burden the insurances enormously but also patients that are not insured or whose insurance does not cover the diagnostic services completely [6]. Besides the direct costs, many affected patients suffer as well financial problems and economic losses due to loss of productivity caused by reduced working times, absence and unemployment [8].

To provide a point of contact for patients and physicians who are at a loss and to find the right diagnosis in a timely manner, interdisciplinary special centers for rare diseases have been established. On the one hand, experts for a wide range of diseases work in these centers who exchange ideas in an interdisciplinary way and have a large base of knowledge and experience, and on the other hand the centers offer all the necessary structures and resources to diagnose and treat patients with rare diseases [9].

Diagnostic decision support systems promise to make diagnosis even easier. Through the use of artificial intelligence, physicians should be supported in their diagnostic considerations [10-12]. The goals of these diagnostic support systems can be different: more accuracy, more effectiveness, less costs, wider distribution and less time [12]. There are tools that specialize in detecting individual rare diseases while others detect rare diseases more in general. According to the different objectives different data sources and methods are considered, what makes the assessment process even more diverse [12].

A retrospective study conducted at the Outpatient Clinic for rare inflammatory systemic diseases at the Hannover Medical School reveals that there is a great potential for diagnostic cost savings through the use of expert diagnostic decision systems. The study shows that diagnostic decision systems can achieve a significant acceleration of the diagnostic process [10].

Based on these findings it is the aim of this study to evaluate the reduction in diagnostic costs incurred, resulting from rapid referral to an expert and from the use of a diagnostic support system for rare diseases.

## Methodology

A retrospective study was conducted in the outpatient clinic for rare inflammatory systemic diseases at the Hannover Medical School.

The outpatient clinic for rare inflammatory systemic diseases is an expert center. The cases we studied (Supplementary Table 1) were assigned to the expert in rare inflammatory diseases with renal involvement.

First, we determined all diagnostic costs incurred in these cases. Secondly, we investigated the potential reduction in diagnostic delay that could be achieved by the use of an DDSS. From these data, we calculated the potential reduction in incurred costs that could be achieved.

The rationale was that if a diagnosis is correctly suggested by a DDSS, targeted diagnostics will enable a rapid diagnosis thus leading to a reduction in costs.

### Case selection

Patient cases from a previous study [10] were selected for the improvement of diagnostic processes through diagnostic support systems. These are patients who have a confirmed diagnosis of a rare disease with a documented date.

Cases with a particularly severe course and diagnostic odysseys were prioritized according to a subjective assessment by the head of the Rare Inflammatory System Diseases Clinic [10]. The prerequisite for participation in the study was a complete medical record with as much documentation as possible of the patient’s visits to the physicians, hospital stays, imaging and laboratory diagnostics over the entire course of the disease.

### Approach

First, the date on which the patient first presented to a physician with disease-specific symptoms was extracted from all files.

All data from subsequent diagnostic examinations related to the disease were also documented in tabular form until the final diagnosis was made. In the tabular overviews it was noted for each date whether it was a physician’s appointment or an inpatient hospital stay. In addition, all diagnostic examinations carried out on the individual dates were recorded in the tables down to the smallest detail. For patients diagnosed by the expert for rare diseases at the Hannover Medical School, the date of first presentation to the expert was also recorded.

There are three ways in which patients come to the expert center. Either as an acute emergency, by referral from a specialist for diagnostic clarification, or patients with an already confirmed diagnosis of a rare disease.

For this reason, the patients were divided into three groups for the economic analyses:

1. Diagnosis by the expert after a long diagnostic odyssey:

This group included patients who had particularly long courses of disease with diagnostic odysseys. Specialists reached their diagnostic limits with patients in this group and therefore referred the patients to the expert, who was finally able to make the correct diagnosis.

2. Diagnosis by the expert in an acute course:

Patients who had particularly severe, life-threatening courses and were referred to the expert as an emergency in a period of less than one month or had to be treated directly by the expert were assigned to this group.

3. Diagnosis by external medical specialists:

If a diagnosis was made outside of Hannover Medical School and the patient was referred to the expert at Hannover Medical School for further treatment due to lack of expertise, the patient was assigned to this group.

### The Diagnostic Decision Support System (DDSS)

The prototype of the knowledge-based, probabilistic diagnostic support system Ada, named Ada DX, was used in the study [10].

First, the point of time with the first disease-relevant findings was recorded for each patient. For each patient the data of all the documented visits at health care providers were extracted from the medical record. Data from written and dated documentations were taken into account, such as medical discharge letters, referral forms and documented test results from laboratories and pathologists. All data were recorded on a year and month basis [10]. From the time of the first disease-relevant findings and symptoms all examination findings, test results and risk factors were retrospectively extracted from the patient files and entered into Ada DX by the expert [10].

The findings were assigned to the time of the respective visit, but not to previous visits based on medical history information. Previously collected findings were retained for future visits if they were not refuted by other documented information [10]. While all the pathological findings were extracted, non-pathological findings were not recorded unless they were important for excluding a differential diagnosis.

With the entry of first symptoms Ada DX constructs two lists:

On the one hand, a list is created in which diseases are arranged according to the fit of the symptom constellations to the specific disease phenotypes and, on the other hand, a “probability disease list” in which diseases are assigned to the symptom according to probabilities taking into account epidemiology [10].

The lists are adjusted with each visit at which new diagnostic findings occur.

To assess the possible impact on the time of diagnosis the disease suggestions in the “fit”- disease list are included. The “fit”- disease list was chosen because it is not expected that rare diseases are diseases with a high probability of occurrence [10].

They are rather diseases which are only considered as a tentative diagnosis which are suggested due to the fit of the constellation of findings [10].

As Ada DX is still a prototype, the system does not yet cover a sufficient number of diseases from the broad spectrum of rare diseases.

At the time of the previous study [10], Ada’s knowledge base contained several hundred rare diseases based on the Orphanet definitions. Diseases that were not yet part of the Ada knowledge base were added to the knowledge base during the study by creating models for missing diseases. The goal of the study was to determine the potential of Ada DX for early detection of rare diseases. Ada DX was chosen for various reasons: transparency of the reasoning, novelty of the engine and interface.

### Economic evaluation

All included patient cases were evaluated individually. This means that medical services provided in connection with the disease were extracted from all patient records since the first signs of the disease appeared. The medical services were tabulated by days and years.

Since our entire patient collective had statutory health insurance, the “Einheitliche Bewertungsmaßstab (EBM)” was used as the basis for our health economic analyses.

EBM is a remuneration system for statutory health care in Germany. With the help of billing numbers, each service provided by a physician is assigned to a specific remuneration.

In the case of patients with statutory health insurance, physicians do not receive their salaries directly from the patient, but bill the Association of Statutory Health Insurance Physicians (Kassenärztliche Vereinigung, KV) for their services according to EBM. The “Kassenärztliche Vereinigungen (KVs)” are corporations under public law that organize medical care through statutory health insurance funds [13].

For our analyses, we used the current online version of EBM as of 01.01.2021. To be able to retrospectively determine the costs incurred, we assigned the corresponding cost parameters based on the EBM to the medical services we tabulated from the individual patient records.

Supplementary Table 2 shows all EBM numbers used in this study to determine the direct cost factors.

Depending on the particular analyses, single or multiple EBM numbers were required to represent the specific level of costs incurred. For the calculation of treatment costs, physician fees for medical services were added according to the billing numbers.

However, there are certain rules for billing. For example, EBM numbers can mean exclusions for other EBM numbers that are billed in the same session. In this case, these cannot be added together. If a particular examination could not be assigned to a single billing number in the EBM pool, a flat rate was calculated based on similar examination methods.

Using the method described above, all direct costs incurred could be calculated retrospectively. Data cleaning, sorting, and manipulation, as well as calculations, were primarily accomplished using R and Excel.

To determine the potential savings of diagnostic decision support systems, the timestamps for the times when AdaDX displayed the correct diagnosis among the top-5 or as the top-1 suggestion were transferred from the study by Ronicke et al. [10] and integrated.

To calculate the total costs that would have incurred if the treating physicians had used a diagnostic decision support system, the fees of all physician services were added that were actually provided up to the date of the hypothetical diagnosis.

## Results

93 patient cases were analyzed. 15 cases were excluded due to missing data. Of the remaining cases, 54 cases were assigned to group one (*“diagnosis by the expert after a long diagnostic odyssey”)*, 8 cases to group two *(“diagnosis by the expert in an acute course”*) and 16 cases to group three (“*diagnosis by external medical specialists”*). Table 1 provides an overview of the cases.

**Table. 1:** Characteristics of included cases

	Female	Male	Total
Number of included cases	48	30	78
Cases with multiple diagnoses	5	4	9
Mean age of onset	39	41	40
Mean age at diagnosis	43	46	44
Average number of visits to the doctor	14 (0 to 63)	9 (0 to 48)	12 (0 to 63)
Average days in inpatient treatment	14 (0 to 56)	25 (0 to 85)	18 (0 to 85)

For the economic analyses of DDSS, 76 of the patient cases could be evaluated:

In 68 included cases the point of time could be documented when Ada DX indicated the correct diagnosis among the top 5 suggestions as well as the point of time when Ada DX put the correct suggestion in the first place of the suggestions.

Five of the included cases had only one hit on the correct top 5 suggestions, but the correct final diagnosis could not be determined by the system.

For the remaining three cases, Ada DX could not come to any correct conclusion.

**Fig. 1:**
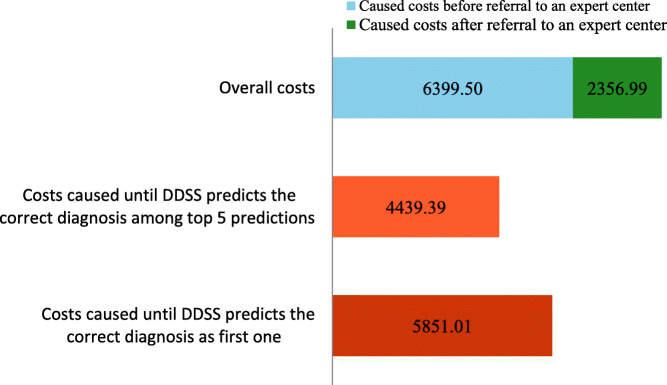
Analysis of the caused costs until a correct diagnosis was achieved (“diagnosis by the expert after a long diagnostic odyssey”; *n* = 54; mean values)

The statistical analysis of the 54 patients of group one (“Diagnosis by the expert after a long diagnostic odyssey”) showed that the patients had to visit an average of 8 different physicians/clinics until they received a correct diagnosis. This was also reflected in the economic analyses:

The economic analysis of group one showed that the total costs incurred until a correct diagnosis was made averaged €8.756,49 (median: €7.025,78). This represents the average amount of total direct costs incurred until a successful diagnosis could be made. Dividing total costs into two distinct sections, subdivided by the date a patient was referred to the expert center, yields a $\frac{3}{4}$ to $\frac{1}{4}$ distribution (Fig. 1). On average, $\frac{3}{4}$ of the total costs had incurred before a patient was transferred to the expert center and $\frac{1}{4}$ of the costs incurred during treatment and successful diagnosis at the expert center.

Based on our calculations, we estimate the cost reductions enabled by a DDSS to be up to 50 %. By the time the correct diagnosis was displayed among the top 5 suggestions, an average of €4.439,39 in physician costs would have been incurred with the help of the DDSS. Until the DDSS suggests the correct diagnosis first, an average of €5.851,01 incurred, which is ~ 67 % of the total costs. During the analysis of the data, it was noticed that certain analyses were repeated several times and at different times in the patient’s medical history.

**Fig. 2:**
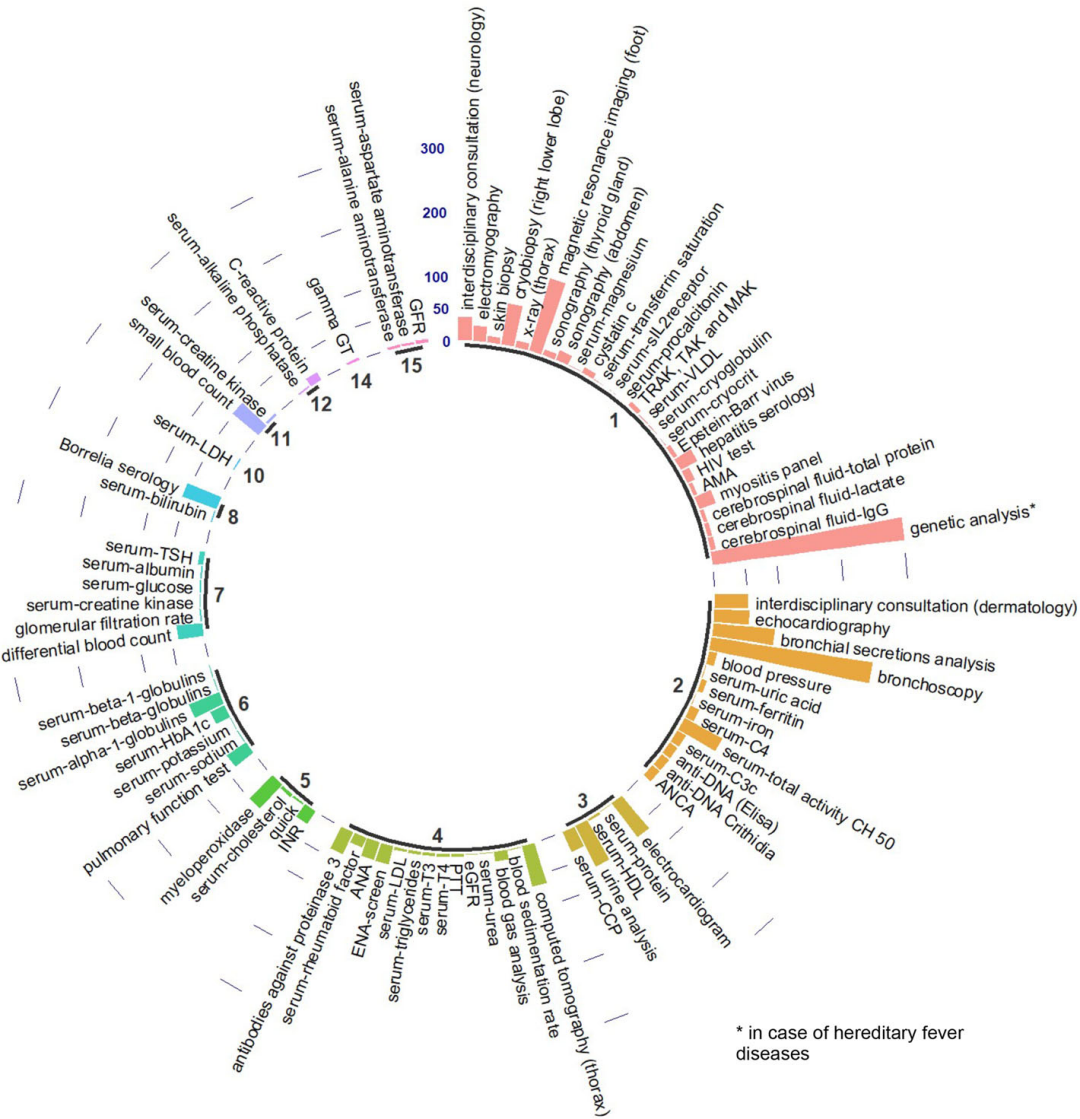
Exemplary distribution of data evaluated from a single representative patient (top). Bold numbers represent the amount of times an analysis was done. Type of analysis is labelled on top of their bars. Length of bars reveals the caused costs (cumulative costs for analyses which were done more than once). Label of caused costs axis is shown on the top centre [€]

The distribution shown in Fig. 2 belongs to the data of a single patient, but is representative for the evaluated group. In total 184 different analyses were done during the entire diagnosis process for this patient. 93 of these analyses were done twice (on two different dates). Two analyses (for this particular patient: alanine aminotransferase and aspartate aminotransferase) were done on 15 different dates. To get an idea of the data, the average, median and maximum number of repetitions per analysis was calculated for group 1. On average, an analysis was repeated on 3 different dates throughout the time documented by the attending physician (mean), whereas the majority of analyses were repeated only twice (median). Few analyses, on the other hand, were repeated an average of 11.47 [1;32] times, while the highest number of an analysis repeated on different dates was found to be 32. Urine and serum tests, blood counts, antibody tests, and colonoscopy and computed tomography were generally repeated more frequently. Genetic analyses, pathogen tests, and cerebrospinal fluid examinations were generally performed once.

**Fig. 3:**
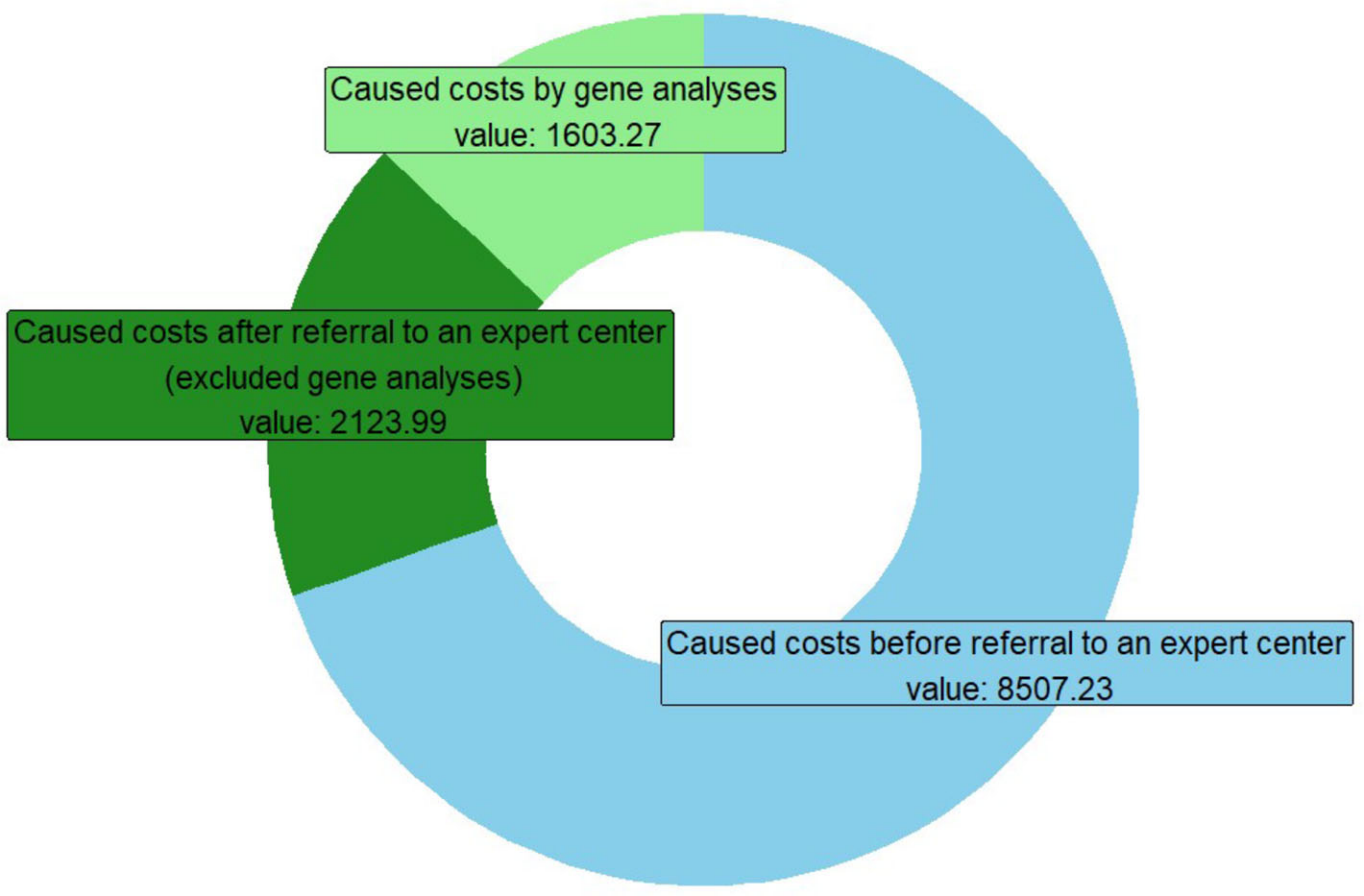
Relation of caused costs by gene analyses to overall costs (n = 15; mean values)

Genetic examinations turned out to be conspicuously cost-intensive in the analyses. For this reason, a sub-analysis of the costs incurred by genetic analyses was created. Genetic testing was almost exclusively initiated by the expert. These sub-analyses of the diagnostic costs of the expert showed that in cases (n = 15) where genetic analyses are performed (Fig. 3), significantly higher costs incur, which even account for about 50 % of the total costs incurred by the expert. In relation to the total costs, human genetic analyses thus accounted for 18 %.

With an average of €12.234,49 in diagnostic costs, cases in which genetic analyses were performed were significantly higher in total costs than the average of study participants.

The small group 2 (n = 8) which included all the patients that had particularly severe, life-threatening courses and had to be diagnosed by the expert as quickly as possible showed a slightly different distribution of the costs.

As a rule, these patients visited on average only 2–3 different physicians/clinics until the correct diagnosis was made, but they still caused relatively high costs.

**Fig. 4:**
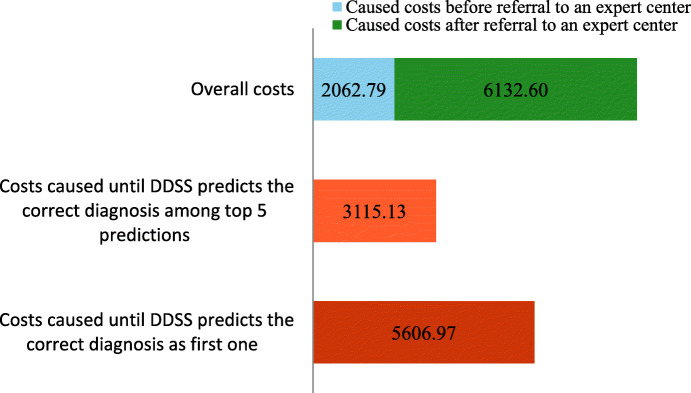
Analysis of the caused costs until a correct diagnosis was achieved (“diagnosis by the expert in an acute course”; n = 8; mean values)

It is noticeable that the majority of the costs incurred occurred after the patients were transferred to the expert center (Fig. 4). This could be due to the fact that the acute condition of the patients made rapid, extensive diagnostics unavoidable. On average, €8.195,39 were spent until the correct diagnosis was found. The amount of costs incurred is similar to groups 1 and 3. However, based on the estimated times when an expert would have made the correct diagnosis using the DDSS, the costs incurred are almost as high as in group 1 and significantly lower than the mean value of the total costs. Predictions of the correct diagnosis among the top 5 were achieved significantly earlier and are associated with lower mean costs of €2.787,87.

Group 3 consisted of 16 patients who were correctly diagnosed outside of MHH and visited an average of 3–4 different physicians/clinics prior to diagnosis. Due to lack of expertise, patients in this group were referred to the expert at MHH for further treatment.

**Fig. 5:**
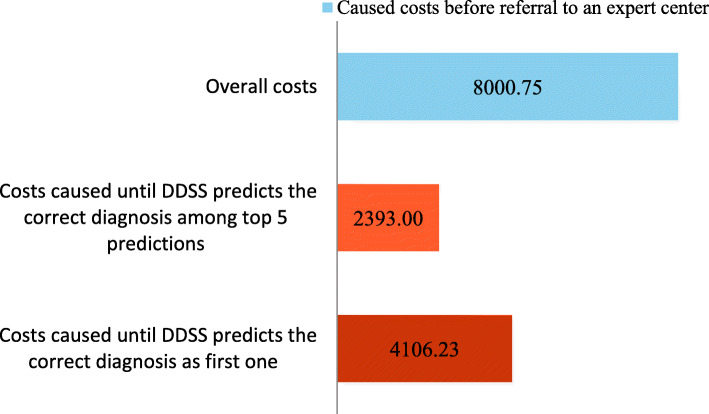
Analysis of the caused costs until a correct diagnosis was achieved (“diagnosis by external medical experts”; n = 16; mean values)

The total costs of €8.000,75 were almost as high as in group 1 and there incurred obviously no costs at MHH (Fig. 5). On average, costs of €4.106,23 would have incurred with the DDSS support until the correct diagnosis was first made. Again, the costs caused were almost 50 % lower compared to the total costs. Among the top 5 predictions, the correct diagnosis appeared earlier and, on average, at a time when the costs caused amounted to €2.393.

Independent of the group evaluated, the mean value of the total costs was about €8.000. The ratio of total costs and the costs that would have incurred if DDSS had been used are also almost the same. The costs incurred until the DDSS first predicts the correct diagnosis range from 51 to 68 % of the total costs. Among the top 5 predictions of the DDSS, the correct diagnosis appears faster, resulting in incurred costs between 30 and 50 % of total costs.

These results show a clear potential for savings through the use of DDSS.

In addition to the benefits of saving time, allowing earlier systematic treatment, which in turn is associated with a higher quality of life for the patient, the potential for cost savings is another benefit that should be further investigated in prospective studies.

## Discussion

The main goal of our study was to examine, if in the case of rare diseases health economic savings can be achieved by consulting an expert and using diagnosis support systems.

### Health economic savings by the expert

In the case of long courses of disease of patients in the first group it can be seen from the results that health economic savings through experts are possible here. Whereas the expert needed €2.356,99 on average for the successful diagnosis in a much shorter time, an average of €6.399,50 incurred in diagnostic costs prior to presentation to an expert.

The saving potential of the expert lies in the fact that they achieve a faster, target-oriented diagnosis through their specialist knowledge and experience.

When evaluating the data on the treatments that took place before the presentation to an expert, various causes were identified that led to delays in diagnosis and thus to considerable costs being incurred. These are briefly described below:

The main problem was the lack of expertise of many medical professionals about rare diseases. In a Belgian study from the year 2019 [4] 86 % of the interviewed general practitioners stated to have insufficient knowledge about rare diseases. This was reflected in most of the cases we examined, because the visit to a general practitioner was usually the first stop of a treatment odyssey. In GP practices laboratory chemical diagnostics, in particular were frequently repeated and many referrals to various specialists followed where extensive diagnostic tests and imaging examinations were carried out hereafter. In this respect the interdisciplinary aspect turned out to be problematic. As already described in other publications [7] the variety of visited physicians led to a less interdisciplinary exchange, a lot of important information went lost and examinations were repeated unnecessarily.

Last but not least the patients changed their physicians during treatment because of dissatisfaction and frustration about the lacking diagnosis which also led to renewed examinations and consequently to renewed cost.

Often false diagnoses were taken over in physician’s letters and not questioned anew which was another reason for delay of diagnosis. The helplessness ended for a large part of the patients at some point in the diagnosis in a mental disorder which then could be refuted by the correct diagnosis. This resulted in additionally immense costs in view of the unnecessary therapeutic interventions and related hospital stays which however are not part of this study.

Also are long waiting times at specialist physicians responsible for diagnosis delays. An insurance survey of KBV (= German Federal Association of Statutory Health Insurance Physicians) of 2020 showed that every third German had to wait more than three weeks for an appointment with a specialist [14].

In comparison a more structured and targeted approach was noticeable in the treatment by the expert. Directly for the presentation all documents about the examinations carried out so far had to be submitted by the patient. Through the review of the documents, it was possible to gather first conclusive, diagnostic information.

Also, the performance tables we prepared for the analyses clearly showed that the expert conducted more extensive investigations. More laboratory parameters were collected than in most of the previous examinations and there was an interdisciplinary exchange. In addition to his/her expert knowledge the location is undoubtedly an advantage for the expert. The connection to the clinic makes imaging examinations possible relatively fast and long waiting times cease through rapid internal consultations.

As 80 % of rare diseases are of genetic origin [15] and a complete decoding of the human genome did not take place until 2003 [16], it must be considered that especially in the older patient cases´, where the genetic analyses were not possible, the expert would likely also have needed more time for the diagnosis. As the health economic analyses of group two with a small number of acute cases, some of which were severe and life-threatening, illustrated it is for the expert not possible in all cases to achieve savings. In these patient cases the first priority was to make a diagnosis as quickly as possible so that an immediate therapy could start. On average an amount of €8.195,39 was used within a very short time for the entire diagnostic of which the expert spent an average of €6.132,60 Euro.

### Health economic savings through the use of diagnostic support systems (DDSS) by experts

The results of the economic analyses point to a saving potential through DDSS. On average, DDSS Ada DX would have caused less costs in diagnostics in all the three groups. The cost savings can be attributed to two reasons:

The first causal reason is that unnecessary repetitions of examinations can be avoided through the use of DDSS as all the examinations are stored in the system.

The second even more important cause of savings is a quicker diagnosis through DDSS. Ronicke et al. [10] could show in the previous retrospective study that the correct diagnosis through Ada DX was hypothetically well before the clinical diagnosis.

The explanation for a quicker diagnosis and thus cost reduction lies in the diagnostic quality improvement through DDSS [17, 18, 20]. The strength of DDSS is that it supports the physician in areas of diagnostics where the human brain reaches its limits in terms of its ability to remember and process information. With 8.000 known rare diseases it is clear that it is impossible for even the most experienced physician to know each disease with all its symptoms [19]. Through their evidence-based clinical knowledge data base DDSS can help with these cognitive barriers and sensitize medical professionals to the presence of a rare disease by suggesting diagnoses.

With the awareness that a patient may be suffering from a rare disease, the GP, for example, as the first contact could consult an expert and thereby increase the diagnostic quality [18], and if necessary, arrange for a faster referral to an expert center. On the whole, this approach would prevent diagnostic odysseys and save immense costs.

It can be concluded that every medical professional can qualitatively improve their diagnostic skills with DDSS [20] and thereby realize savings. However, in comparison, an expert will take a much more targeted approach due to their expertise in anamnesis and diagnostics and can interpret the suggestion of DDSS better through experience and so exclude false-positive results more quickly [10]. Consequently, a more cost-efficient diagnosis can be expected from an expert.

The condition necessary to benefit from the above advantages of DDSS is to record as many diseases as possible and to permanently develop the knowledge base further according to the latest scientific findings. However, should a decision-making tool not be sufficiently developed and use outdated literature, it represents more a security risk than a benefit [21].

Also, an optimal integration into the stressful clinic and practice every day work is of great importance for fully utilizing the efficiency of DDSS [21]. A simple application which is as close as practicable to the core functionality of the already existing management system, is desirable here [21, 22]. Furthermore, training on the correct use of DDSS should be provided, as lack of knowledge can hinder the effective use of DDSS [22, 23].

After the saving potential through DDSS has now been widely explained, it must finally be mentioned that in addition to the possible training costs also costs occur for the integration and installation of DDSS [24].

Taking an overall view of the most analyses that were examined in this study, the median costs tended to be below the mean value indicating that a small proportion of the patients with high costs have driven up the average costs resulting in a positive imbalance. The typical (median) patient caused in all likelihood lower costs than indicated here while patients with especially long odysseys caused above-average costs, what was to be expected.

### Limitations of the study and future points of contact

In principle, a retrospective approach is suitable for the studies of economic benefits through expert knowledge and DDSS. A disadvantage of the retrospective approach is that an evaluation of the results is only explorative.

The patient cases we studied are only a small grouping of special rare diseases, which is why the results cannot be applied to all rare diseases.

There are some disadvantages in data entry due to the retrospective study design:

Although great care was taken to ensure the completeness of the patient files, it must be assumed that in some cases, due to the overall very long course of the disease documentations of medical examinations that were made before presentation to the expert, are missing. It can thus be presumed that the costs can even be higher before expert presentation.

Although case entry was based on chronological entry of written documented information from medical records to reduce retrospective bias and retrospective misinterpretation, it was not blinded because patient cases were already known [10].

Another limitation due to the retrospective study design is that the effects of the diagnostic support system were not measured in clinical practice. Some authors are concerned that there may be increased clinical testing in inexperienced clinicians in clinical practice [25]. However, Elkin et al. show that the use of diagnostic decision support systems in everyday clinical practice tends to result in less testing and more efficiency [17].

As the evidence increases, the probability differences between the diseases in the lists grow. However, it must be noted here that the responsibility for determining when a diagnosis can be considered correct lies with the treating physician. Accordingly, the suggestion of a correct diagnosis by the system does not mean that it will be diagnosed. Therefore, diagnostic time gains and cost savings from the system may vary by physician.

As far as the economic analyses are concerned, our results are limited to costs incurred in Germany. Viewed internationally, for example in comparison with the USA, the diagnostic costs can be many times higher. An international comparative study would certainly be of interest.

The patient collective comprised patients from all over Germany, some of them who travelled far to be examined by the experts. This offers potential for a study on indirect costs, such as those caused by journeys to physicians or productivity restrictions that arose in the course of the treatment odysseys. Angelis et al. showed that depending on the type of rare disease massive indirect costs can also arise, which can even exceed the direct costs [8]. In addition, fiscal cost implications resulting from reduced tax payments and public income support, which often arise in the case of disability or reduced earning capacity due to rare diseases [26], could be investigated in the future.

It would also be of interest to determine the impact of a correct diagnosis on patients’ quality of life in a potential study.

## Conclusions

The study showed that in case of rare diseases high diagnostic costs incur. The later the referral to an expert the more extreme became the economic burden. With the help of expert knowledge, the diagnostic processes could be accelerated and unnecessary examinations avoided.

Ada DX provided correct suggestions for rare diseases before clinical diagnosis in most patient cases which was reflected in the economic analyses with a high potential for savings.

DDSS not only saves costs through earlier diagnosis, but also by avoiding unnecessary repeat examinations. It must be considered that system implementations require expenditure - but this should be compensated for by the expected benefits.

In conclusion, based on this study, rapid referral to an expert and the use of a DDSS promise a cost-effective, faster diagnosis in rare disease patients.

## Supplementary information

**Additional file 1**

Additional file 2 Supplementary Table 2

## Data Availability

The data analyzed during this study are included in this published article and its supplemental information files. The data supporting the results of this study are available on request from the corresponding author TV. The data are not publicly available because they contain information that could compromise study participant privacy and/or consent.

## Abbreviations

DDSS: Diagnostic decision support systems
EBM: The Uniform Evaluation Scale
KV: Association of Statutory Health Insurance Physicians
MHH: Hannover Medical School
GP: general practitioner
