# Supplementary material for: Health economic benefits through the use of diagnostic support systems and expert knowledge
Source: BMC Health Serv Res. 2021 Sep 9;21:947. doi: 10.1186/s12913-021-06926-y (PMC8431907; doi:10.1186/s12913-021-06926-y)
Supplement: Supplementary file 2 — Supplementary Table 2 [file 12913_2021_6926_MOESM2_ESM.docx]

*Supplemental Table 2:* Listing of all used UAS (EBM) numbers to calculate direct costs.

| 10343 | 31675 | 32785 | 01602 | 32083 |
| --- | --- | --- | --- | --- |
| 13211 | 31684 | 32839 | 01727 | 32084 |
| 13400 | 31685 | 32865 | 01756 | 32092 |
| 13421 | 31697 | 33012 | 01766 | 32094 |
| 13425 | 31698 | 33020 | 01833 | 32112 |
| 13431 | 31714 | 33023 | 01930 | 32113 |
| 13545 | 31718 | 33030 | 01935 | 32120 |
| 13700 | 31719 | 33050 | 02101 | 32121 |
| 13701 | 31821 | 33061 | 02200 | 32122 |
| 16310 | 31822 | 33070 | 02300 | 32125 |
| 16321 | 31823 | 33072 | 02312 | 32128 |
| 16322 | 31824 | 34221 | 02342 | 32135 |
| 17311 | 31825 | 34223 | 02343 | 32248 |
| 17320 | 31827 | 34231 | 02360 | 32267 |
| 17330 | 32042 | 34232 | 02401 | 32270 |
| 18700 | 32104 | 34233 | 03242 | 32353 |
| 21211 | 32124 | 34234 | 03321 | 32354 |
| 21218 | 32150 | 34255 | 03330 | 32355 |
| 24211 | 32163 | 34270 | 04220 | 32356 |
| 30110 | 32166 | 34291 | 04221 | 32357 |
| 30111 | 32207 | 34310 | 04528 | 32372 |
| 30122 | 32237 | 34320 | 05330 | 32411 |
| 30900 | 32247 | 34321 | 05341 | 32413 |
| 30952 | 32251 | 34330 | 06211 | 32461 |
| 31121 | 32352 | 34342 | 06225 | 32463 |
| 31124 | 32385 | 34351 | 09315 | 32489 |
| 31134 | 32386 | 34410 | 09317 | 32490 |
| 31135 | 32426 | 34440 | 09361 | 32492 |
| 31142 | 32435 | 34441 | 126220 | 32493 |
| 31146 | 32448 | 34442 | 13662 | 32560 |
| 31232 | 32491 | 34486 | 13663 | 32564 |
| 31237 | 32494 | 34600 | 19319 | 32566 |
| 31257 | 32503 | 34601 | 19404 | 32587 |
| 31292 | 32540 | 34701 | 32035 | 32589 |
| 31312 | 32542 | 35600 | 32036 | 32632 |
| 31333 | 32569 | 36333 | 32037 | 32670 |
| 31501 | 32575 | 36504 | 32038 | 32681 |
| 31503 | 32586 | 36823 | 32039 | 32703 |
| 31504 | 32598 | 36881 | 32047 | 32706 |
| 31505 | 32601 | 40351 | 32051 | 32721 |
| 31507 | 32606 | 01602 | 32056 | 32722 |
| 31611 | 32610 | 13391 | 32064 | 32780 |
| 31614 | 32623 | 32058 | 32065 | 32823 |
| 31615 | 32626 | 32081 | 32066 | 33022 |
| 31618 | 32629 | 32461 | 32067 | 33042 |
| 31619 | 32723 | 32575 | 32069 | 33043 |
| 31658 | 32724 | 34410 | 32070 | 34220 |
| 31659 | 32725 | 01321 | 32071 | 34341 |
| 31662 | 32726 | 01600 | 32074 | 34421 |
| 31663 | 32784 | 01601 | 32082 |  |
